# COVID-19 and the Brain: The Neuropathological Italian Experience on 33 Adult Autopsies

**DOI:** 10.3390/biom12050629

**Published:** 2022-04-25

**Authors:** Viscardo P. Fabbri, Mattia Riefolo, Tiziana Lazzarotto, Liliana Gabrielli, Giovanna Cenacchi, Carmine Gallo, Raffaele Aspide, Guido Frascaroli, Rocco Liguori, Raffaele Lodi, Caterina Tonon, Antonietta D’Errico, Maria Pia Foschini

**Affiliations:** 1 Department of Biomedical and Neuromotor Sciences, University of Bologna, 40139 Bologna, Italy; giovanna.cenacchi@unibo.it (G.C.); rocco.liguori@unibo.it (R.L.); raffaele.lodi@unibo.it (R.L.); caterina.tonon@unibo.it (C.T.); mariapia.foschini@unibo.it (M.P.F.); 2 Department of Experimental, Diagnostic and Specialty Medicine, Azienda Ospedaliero-Universitaria di Bologna, Via Albertoni 15, 40138 Bologna, Italy; mattia.riefolo@unibo.it (M.R.); tiziana.lazzarotto@unibo.it (T.L.); antonietta.derrico@unibo.it (A.D.); 3 Operative Unit of Clinical Microbiology, Azienda Ospedaliero-Universitaria di Bologna, Via Albertoni 15, 40138 Bologna, Italy; liliana.gabrielli@aosp.bo.it; 4 Unit of Anatomic Pathology, Department of Oncology, Bellaria Hospital, Via Altura 3, 40139 Bologna, Italy; c.gallo@ausl.bologna.it; 5 Anesthesia and Neurointensive Care Unit, IRCCS Institute of Neurological Science of Bologna, Via Altura 3, 40139 Bologna, Italy; raffaele.aspide@isnb.it; 6 Intensive Unit Care, Azienda Ospedaliero-Universitaria di Bologna, Via Albertoni 15, 40138 Bologna, Italy; guido.frascaroli@aosp.bo.it; 7 IRCCS Istituto delle Scienze Neurologiche di Bologna, Via Altura 3, 40139 Bologna, Italy; 8 Functional and Molecular Neuroimaging Unit, IRCCS Istituto delle Scienze Neurologiche di Bologna, 40139 Bologna, Italy; 9 Unit of Pathology, Azienda Ospedaliero-Universitaria di Bologna, Via Albertoni 15, 40138 Bologna, Italy

**Keywords:** COVID-19, neuropathology, endovascular microthrombi, hypoxic-ischemic injury, bacterial superinfection, microglia activation, astrogliosis, chronic inflammation

## Abstract

Neurological symptoms are increasingly recognized in SARS-CoV-2 infected individuals. However, the neuropathogenesis remains unclear and it is not possible to define a specific damage pattern due to brain virus infection. In the present study, 33 cases of brain autopsies performed during the first (February–April 2020) and the second/third (November 2020–April 2021) pandemic waves are described. In all the cases, SARS-CoV-2 RNA was searched. Pathological findings are described and compared with those presently published.

## 1. Introduction

The coronavirus disease 2019 (COVID-19) is caused by the Severe Acute Respiratory Syndrome Coronavirus 2 (SARS-CoV-2) and it was declared pandemic by the World Health Organization (WHO) in March 2020 [1], after its outbreak in Wuhan, China.

In Italy, as of April 2021, more than 15 million persons have been diagnosed with COVID-19 and 160,000 of them have died [2].

Most patients with COVID-19 are asymptomatic or have mild respiratory symptoms (fever, cold, cough) but about 20% develop a moderate to severe illness characterized by interstitial pneumonia, acute hypoxemic respiratory failure and, rarely, multiple organ dysfunction syndrome (MODS) [3].

It is now well understood that severe COVID-19 is not limited to the respiratory tract, but complications involving blood homeostasis [4] and other organs (such as heart, liver, kidney, skin) have been described [5,6,7,8].

Retrospective series focused on hospitalized patients have also reported several types of neurological manifestations in COVID-19, ranging from headache, myalgia, smell and taste disturbances (without significant nasal obstruction) to Guillain–Barre syndrome, encephalopathy, myelopathy, encephalitis and acute cerebro-vascular events [9].

In a recent study [10] we investigated the neuroinvasive potential of SARS-CoV-2, searching for morphological and microbiological features of viral involvement of the central nervous system (CNS) in individuals who died of COVID-19 related respiratory failure during the first pandemic wave (February–April 2020).

SARS-CoV-2 RNA was retrieved from the olfactory bulbs and tracts and from the brain tissue in 1 out of 11 patients only, while most of the brain damage was related to thrombosis.

During the second and the third pandemic waves (November 2020–April 2021), a further 22 brain autopsies were performed. SARS-CoV-2 RNA was searched in all cases and transmission electron microscopy (TEM) was also added to detect the viral particles on positive specimens.

Aim of the present paper is to report the pathological findings observed in the brain autopsies in the first, second and third pandemic waves.

## 2. Materials and Methods

### 2.1. Study Setting and Data Collection

Complete postmortem examinations were performed according to standard procedures on all 33 deceased patients with diagnosis of SARS-CoV-2 related pneumonia (comprising 10 patients previously reported, 30). The diagnosis of SARS-CoV-2 infection was performed by RT-PCR on pre mortem oropharyngeal swabs and confirmed on post mortem lung tissues in 33 patients. In one case SARS-CoV-2 pneumonia was diagnosed after autopsy (case 29). Autopsies were performed on patients with an atypical clinical course in order to identify relevant pathological features and to provide clinicians with an appropriate feedback. Autopsies were performed in Bologna, Italy, at Bellaria Hospital (Unit of Pathological Anatomy, “M. Malpighi”) and at St. Orsola-Malpighi Polyclinic (Unit of Pathological Anatomy).

### 2.2. Brain Cutting and Gross Examination

Neuropathological gross evaluation was performed, as previously described [10]. Briefly, samples from a standard set of brain regions were obtained as follows: the whole brain stem, basal ganglia, thalamus hippocampus, pre-rolandic and post-rolandic gyrus, frontal (parasaggital), temporal (T2) and occipital lobes (calcarine sulcus). In addition, the olfactory bulbs and tracts up to the lateral olfactory stria were removed. Formalin fixed blocks were paraffin embedded according to routine procedures (FFPE).

### 2.3. Histopathological Examinations

Sections (5 μm thick) were prepared from formalin fixed paraffin embedded blocks (FFPE) and were stained with hematoxylin and eosin (H&E) and Luxol fast blue (LFB). Immunohistochemistry was performed in an automated stainer (Ventana, Tucson, AZ, USA, using Ventana purchased pre-diluted antibodies): NeuN (as neuronal marker), NF (neurofilaments, as axonal marker), GFAP (glialfibrillary acid protein, as glial marker), CD68/CD45 (as microglial markers), CD45/CD20/CD3/CD4/ CD8 (lymphoid markers).

### 2.4. Microbiological Analysis

SARS-CoV-2 RNA was searched in all the cases on sections from olfactory bulb and tract, postrolandic cortex and rostral medulla as previously described (10). FFPE 10 micron sections were pre-treated using 160 µL Deparaffinization Solution (Qiagen, Hilden, Germany) with 180 μL of tissue lysis buffer (ATL buffer, Qiagen) and 20 μL of protease (Proteinase K solution, Qiagen, Germany). Tissues were incubated overnight at 56 °C and one-hour at 90 °C. Then extraction and amplification of nucleic acids were performed using ELITeInGenius^®^ instrument with ELITeInGenius SP 200 and SARS-CoV-2 ELITe MGB Kit (ELITechGroup, Milano, Italy). The tissue viral load was reported as number of copies/microgram RNA. Quantification of RNA was performed using Qubit Assay (Invitrogen by ThermoFisher). Positive results below the lower limit of quantification (2 copies/reaction) were reported as <15 copies/microgram RNA. The identification of key mutations in SARS-CoV-2 Spike Protein gene (E484K, N501Y, HV69-70 del, L452R, K417T, K417N, W152C) was performed with Allplex SARS-CoV-2 Variants I and II Assay, Seegene.

### 2.5. Transmission Electron Microscopy (TEM)

From paraffin blocks of brain section with known SARS-CoV-2 RNA positivity, 5 mm^3^ areas were selected for TEM examination. Tissues were dewaxed in xylene, subsequently washed in a graded series of ethanol (100%, 95%, 70%), rehydrated rapidly in distilled water and then rinsed in cacodylate buffer 0.15 M overnight. Rehydrated tissue samples were postfixed in 1% OsO_4_ in cacodylate buffer, dehydrated in graded ethanol, and embedded in Araldite. Ultrathin sections, stained with uranyl acetate and lead citrate, were examined with Philips TEM CM100 Transmission Electron Microscope.

## 3. Results

### 3.1. Clinical Data 

The present series included 11 patients who died during the first pandemic wave (from February 2020 to April 2020), 9 after the second pandemic wave (from November 2020 to December 2020) and 13 after the third wave (January 2021 to April 2021).

Cases include 25 men and 8 women, ranging in age from 44 to 90 years old (median age: 61).

Twenty-eight patients died after mechanical ventilation support in the intensive care unit or non-intensive care ward; patient 29 died of a sudden cardiac arrest. 

The average length of symptoms duration before death was 20 days (ranging from 5 to 51 days).

On anamnesis the following conditions were present: hypertension (20 cases), obesity (17 cases), previous cerebrovascular incident (2 cases), diabetes (9 cases), dyslipidemia (8 cases), kidney failure (5 cases), ischemic heart disease (3 cases), previous cancer (3 case), chronic obstructive bronchitis (3 cases), idiopathic pulmonary fibrosis (1 case), Crohn’s disease (1 case) and distal arteriopathy (1 case). The most common symptoms at onset were dyspnea and fever (13 cases). Patients 9 and 19 came to the hospital for urinary retention, fever, syncope and may have acquired SARS-CoV2-pneumonia during hospitalization; patient 10 complained of progressive muscular weakness in addition to fever; patient 20 was hospitalized for an isolated syncope. Symptoms related to intracranial hypertension were reported in 4 cases (cases 6, 9, 10, 29); meningitis was clinically suspected in patient 20. No other acute neurological symptoms were clinically evident before death. 

Clinical data are summarized in Supplementary Files—Table S1.

### 3.2. Brain Cutting 

**Figure 1:**
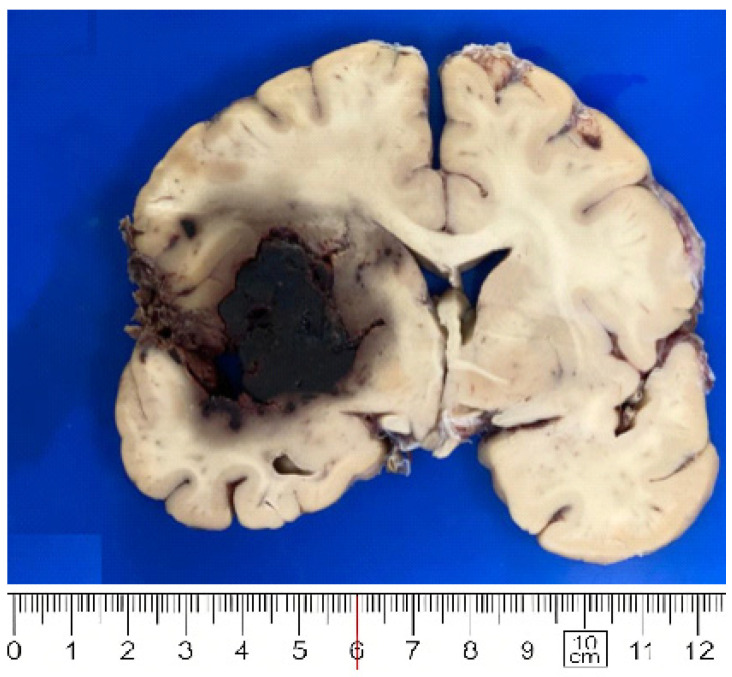
Large hemorrhage in basal ganglia (case 23).

In all cases, the brain surface was edematous with widened gyri, flattened surface, narrowed sulci and leptomeningeal vessels congestion. Brain weight ranged from 1270 to 1870 gr. (mean 1445 gr.). In 4 cases uncal herniation was identified (cases 5, 6, 13, 29). Areas of cerebral infarction were noticed in 6 cases (case 1 left frontal, case 2 right parietal, 3 right frontal, 9 right parietal, 19 right pre-rolandic and 21 pre-rolandic and frontal). Intraparenchymal acute hemorrhage areas were seen in basal ganglia, cortex and pons in case 23 (Figure 1). Meninges were grossly congested: purulent accumulation on the leptomeningeal vault (case 8 and case 11) and focal subarachnoid hemorrhage (case 9, case 23) were observed.

All details are sumarized in Supplementary Files—Table S2.

### 3.3. PCR Results 

RT-PCR detected SARS-CoV-2 RNA in the brain tissues, in 2 cases only (cases 1 and 20). Case 1 was positive in the olfactory tract, in the hyppocampus and in the medulla oblongata. The viral load was 252 copies/microgram RNA. No key mutations in SARS-CoV-2 Spike Protein gene were detected suggesting the presence of original Wuhan-Hu-1 strain of SARS-CoV-2 (wild-type strain). Case 20 revealed positivity in olfactory tract only, with a viral load below the lower limit of quantification (<15 copies/microgram RNA). The low viral load did not allow the identification of key mutations. Patient 1, a 51-year-old man, with a history of drug abuse, had experienced a previous brain infarct, involving the left side motor area, therefore suffering of right hemiplegia. The same patient was in dialysis for chronic renal failure. He had the shortest COVID-19 duration, as he died 6 days after the symptoms’ onset. In addition, in the same case, SARS-CoV-2 RNA was isolated from multiple organs (lungs, liver, kidney and heart). Patient 20 was an 86-year-old man, with a history of chronic renal failure, ischemic cardiomyopathy, diabetes and distal arterial occlusive disease (DAOD). He died 11 days after the symptoms’ onset. 

### 3.4. Histology

On histology (all features are sumarized in Supplementary Files—Table S3), the SARS CoV-2 positive olfactory bulbs, tracts and brain tissues did not differ from the negative ones: they lacked features suggestive of direct viral damage as lymphocytic infiltration, and neuronophagia.

Neuropathological examination revealed several features that can be grouped as follows: microthrombi with recent ischemic damage, acute hemorrhages, global hypoxic-ischemic injury and old ischemic damage.

**Figure 2:**
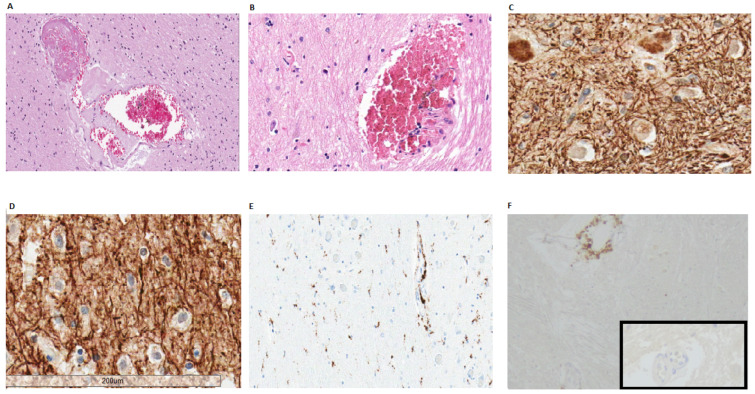
(**A**) Micro-thrombi in small intra-parenchymal vessels (case 28, frontal cortex); (**B**) Micro-hemorrhages surrounding small intraparenchymal of the brain stem (case 13); (**C**,**D**) Neurofilament staining showed no clear axonal damage in the medulla oblongata (**C**) and in the olfactory tract (**D**) of SARS-CoV-2 positive specimens; (**E**) Microglial activation and perivascular histiocytes were evidenced by CD68 (case 1). (**F**) Immunostaining for CD4 highlighted sparse perivascular lymphocytes CD4+ (case 4, medulla oblongata). Inset: normal brain parenchymal vessel without inflammation (immunostaining for CD4).

*Microthrombi with acute and subacute ischemic damage*: all 11 cases (100%) of the first pandemic wave showed a variable number of microthrombi located in the lumen of small intraparenchymal blood vessels (Figure 2A). Focal microscopic (usually 1–2 mm in size) cortical or deep-seated (located in the basal ganglia and through the brainstem) recent infarcts were identified: they consisted of ill-defined pale areas with ischemic red neurons and scattered macrophages, sometimes clearly related to small vessel occlusion by microthrombi. Case 10 showed a larger ischemic damage of the para-hippocampal region whereas cases 2 and 9 were characterized by well-defined lesion with macrophages infiltration, hypertrophy and hyperplasia of capillary vascular cells, plump astrocytes. In the second and third waves small infarcts caused by microthrombi were less frequent (8 out of 22 cases, 38%). In all 33 cases (100%) small blood vessels ectasia, variable perivascular edema, perivascular micro-hemorrhages and scattered hemosiderin-laden macrophages (Figure 2B) were noticed. Necrotic blood vessels or intense perivascular inflammation were not identified. Immunohistochemical analysis (CD20, CD3, CD4, CD8 and CD68) highlighted mild lymphocytic or histiocytic accumulation around occasional vessels (Figure 2C). LFB and NF demonstrated only a slight perivascular myelin reduction with no clear evidence of axonal injury. Activation of microglia and astrocytes was noticed mainly in the brainstem (Figure 2D): transformation from cells with delicate processes to elements with thicker cell projections was detected by immunohistochemistry (CD68 and GFAP). No evidence of neuronophagia was present.

*Acute hemorrhage* was observed in 2 cases (one in each first and second pandemic waves). Case 23 showed multiple acute hemorrhages (ranging from 6 cm to 0.5 cm) through infratentorial leptomeninges, basal ganglia, pons and cortex. No amyloid deposition or septic emboli could be noticed. In this brain microthrombi and subacute infarction were also present. In case 9 supratentorial hemorrhagic areas were seen.

*Global hypoxic-ischemic injury* was observed in all brains (in the first, second and third pandemic waves). Ischemic red neurons were present through all the brain stem, the hippocampal CA1 region and the cerebellar Purkinje cells, consistent with global ischemic injury. 

*Old ischemic damage* (consistent with anamnestic data) was observed in 2 cases. Case 1 showed a 2 cm cystic lesion involving the internal capsule and atrophy of the pyramidal tract, consistent with the history of remote brain infarct. Case 3 presented an area of astrogliosis in the right insula, consistent with an ischemic damage diagnosed 2 years prior death.

Cases 9, 19, 21, 23, 29 and 30 showed atherosclerosis involving bilaterally the internal carotids and the basilar artery.

Internal carotid artery aneurysm was evidenced in case 18.

In addition, 16 out of 33 cases (48%) showed leptomeningeal mild chronic inflammation.

Leptomeningeal inflammation was noticed especially in specimens from the third wave (9 out of 13, 69%).

The most evident features of bacterial superinfection were observed in cases 8, 9 and 11, all observed during the first pandemic wave. Cases 8 and 11 showed macroscopically evident purulent leptomeningeal accumulation and revealed, on histology, endovascular thrombi composed of dense fibrin with neutrophils and necrotic debris, as seen in suppurative leptomeningitis. In case 9 histological evidence of initial infection was detected, characterized by endoluminal leukocytes accumulation and fibrin deposits with blood extravasation. In those patients (cases 8, 9 and 11) Pseudomonas aeruginosa, Candida albicans, Staphylococcus capitis, Staphylococcus aureus, Methicillin-resistant Staphylococcus aureus (MRSA) and Enterococcus faecium were isolated from blood cultures.

Differences between the main three pandemic waves are summarized in Table 1, according to clinical, gross and microscopic features.

### 3.5. Transmission Electron Microscopy (TEM)

**Figure 3:**
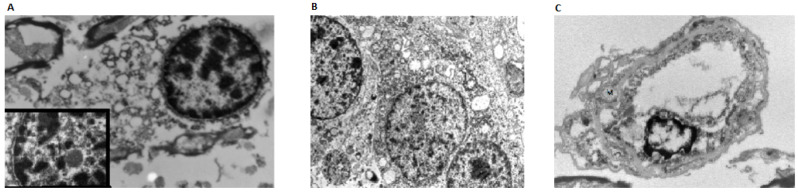
(**A**) TEM analysis in SARS-CoV-2 positive medulla oblongata showed cytoplasmatic vacuoles sized 180 to 211 nm. A nuclear body can be detectable (inset); (**B**) Neuronal cell with two synaptic structures (left) in the setting of a ganglioglioma for comparison; (**C**) a capillary shows severe artifactual alterations without viral-related structures: mitochondria (M) are swollen with fragmented cristae.

In positive brain samples of case 1, neurons showed perinuclear cytoplasmic vacuoles ranging in size from 180 to 211 nm and surrounded by a double membrane (Figure 3A). Occasional small vesicles bulging into the vacuole lumen were seen. In addition, cytoplasmic ribosome aggregates and nuclear bodies were seen, suggestive of unspecific viral infection (Figure 3B). No true spike-delimited virions were detected. The vascular structures showed severe artifactual alterations likely due to suboptimal formalin fixation of autoptic specimens. No specific signs of viral infections were detectable and mitochondria appeared swollen with fragmented cristae (Figure 3C).

## 4. Discussion

Several neurological symptoms have been described in SARS-CoV-2 infected patients, while the exact tissue damage mechanism is still under debate. The main question is to determine if SARS-CoV-2 virus can directly infect the brain parenchyma.

In the present series, SARS-CoV-2 RNA was present in olfactory nerve and in the brain tissue of 2 (out of 33 tested, 6%) patients, but on histology, the PCR positive olfactory tract and brain tissues did not show any specific histological change suggestive of direct viral damage.

TEM analysis showed some feature indicative but non-specific of viral infection.

The small percentage of SARS-CoV-2 infected brains in the present series could be consequent of the method of virus detection, performed on FFPE and not on freshly frozen tissues. Mukerji and Salomon reviewed the COVID-19 brain autopsies published [10,11,12,13,14,15,16,17,18,19,20,21,22,23,24,25,26,27,28,29,30] exploring all the ancillary tests used to detect SARS-CoV-2 in CNS specimens: immunohistochemistry (IHC) identified the viral nucleocapsid (N) or spike (S) proteins in 21% of cases tested (*n* = 16/74); TEM reported virus-like particles in only one case [29]; in situ hybridization (ISH) for viral RNA was negative in the single case tested [27]; RT-PCR identified SARS-CoV-2 RNA in 41% of formalin-fixed paraffin-embedded brain tissue (*n* = 34/84) and in 25% of olfactory bulb/tract specimens (*n* = 9/36) but cannot specifically identify the localization of virus.

Due to the different methods used it is difficult to exactly assess the rate of SARS-CoV-2 brain infection, nevertheless, all the data published confirm that the SARS-CoV-2virus can infect the brain tissue.

Most probably SARS-CoV-2 invade the brain through the Angiotensin-Converting Enzyme 2 (ACE2) receptor of the olfactory epithelium [31] or through endothelial cells of the blood–brain barrier (BBB) with the ACE2 receptor-mediated pathway [31]. In spite of SARS-CoV-2’s ability to invade the brain tissue, most of the brain damage observed is related to intraparenchymal thrombosis and ischemic damage.

The present data are consistent with those found previously published [29,30].

Accordingly, microglial activation and astrogliosis in the brainstem (*n* = 73), acute hypoxic injury from mild to severe (*n* = 59), infarcts/ischemic necrosis (*n* = 22), focal microhemorrhages (*n* = 23), intravascular microthrombi (*n* =12) and neutrophilic plugs (*n* = 3) constituted the most frequently detected brain alterations. T-cell predominant lymphocytic infiltrates can be identified, but without clear evidence of vasculitis or meningoencephalitis [29,30]. In the present series, microthrombi and microscopic infarcts were less frequently detected (8/21, 38%) in cases from the second and the third pandemic waves (November 2020 to April 2021) than in cases of the first pandemic wave (11/11, 100%). This difference can be explained by the more extensive application of anticoagulant therapy in critical patients recently adopted.

From a clinical point of view, different central acute neurological manifestations in patients with COVID-19 have been reported. Ischemic stroke, sinus venous thrombosis and cerebral hemorrhage have been described [32,33,34] and have been related to the inflammation, hypoxia, immobilization or diffuse intravascular coagulation [32]. Our findings and the other neuropathological data in the literature seem to confirm this hypothesis. Dizziness, impaired consciousness, confusion, headache, syncope and seizures have also been reported in SARS-CoV-2 infected patients [35]: they are extremely non-specific and could be the manifestation of an ischemic event, such as global cerebral hypoxia, inflammatory status or electrolyte imbalances rather than a direct viral damage of circumscribed cerebral areas.

All these histological data indicate that CNS is probably “a victim more than a target” in SARS-CoV-2 [36]: intravascular microthrombi and microinfarcts are in keeping with the hypercoagulable state of severe SARS-CoV-2 infected patients whereas the hypoxic-ischemic general condition, related to the respiratory failure, may indeed be worsened by the consequent brainstem damage appearing as a final event.

Clinical and radiological data suggest that SARS-CoV2 can cause meningitis and encephalitis.

The first case of meningoencephalitis was reported in a 24-year-old male with fever and seizures: SARS-CoV-2 RNA was found in the cerebrospinal fluid (CSF) but not in the naso-pharyngeal swab [37]. Duong et al. reported meningoencephalitis in a 41-year-old female who presented with fever and stiff neck [38]: she was tested positive for COVID-19 but the presence of virus RNA in the CSF was not confirmed. Lewis et al. in a recent review highlighted that detection of SARS-CoV-2 in CSF via PCR or evaluation for intrathecal antibody synthesis appears to be rare: of 58 patients whose CSF was tested for SARS-CoV-2 antibody, 7 (12%) had positive antibodies; of 132 patients who had oligoclonal, only 3 (2%) had evidence of intrathecal antibody synthesis; of 77 patients tested for autoimmune antibodies in the CSF, 4 (5%) had positive findings [39].

In our series we did not find signs of viral meningitis but purulent leptomeningitis was seen in 2 cases and bacteria (Pseudomonas aeruginosa, Candida albicans, Staphylococcus capitis, Staphlococcus aureusand Methicillinresistant Staphylococcus aureus, Enterococcus faecium) were isolated, suggesting hematogenous bacterial spread leading to acute bacterial meningitis or encephalitis.

Neuropathological evidence is still not available for Guillain–Barre syndrome [40], Miller–Fisher syndrome [41] or polyneuritis cranialis [42] in SARS-CoV-2 infected patients.

Even more complicated is the debate about subsequent development of chronic neurological disease in COVID-19 patients. This hypothesis is based on experimental models for SARS-CoV and MERSCoV (that already demonstrated the presence of these viruses inside the nerve cells, with degeneration and neuronal death) [43].

Specifically, the evidence from murine lung injury models by coronaviruses emphasizes the correlation between the NLRP3 inflammasome-mediated and the pathological accumulation of neurodegeneration-associated peptides such as fibrillar amyloid-β [44], leading to Alzheimer’s disease.

Much remains also to be learned about the effects of central nervous system chronic inflammation and microenvironment changes after COVID-19 [45].

SARS-CoV-2 infection might elicit the proinflammatory microglia phenotype, which can present in a patient as a neurodegenerative disorder [46].

The presence of reactive pro-inflammatory microglia can also increase the expression of genes that cause neuroinflammation [47].

In the cases here studied, inflammation was minimal to mild: rare T-lymphocytes and macrophages are detected mainly by immunohistochemistry around intraparenchymal vessels or leptomeninges.

In addition, inflammation could have been reduced by long immunosuppressive therapy (steroid). Astrogliosis and microglia activation (sometimes with microglial nodules) are instead prominent.

The present study has some limitations, including the small sample size and the absence of pre-mortem specific neurologic symptoms. Prospective studies are needed to investigate potential correlations between acute, sub-acute, chronic COVID-19 infections and long-term neurological complications.

## 5. Conclusions

The data here shown are consistent with data presently published and features indicate that:

SARS-CoV-2 can directly infect the CNS even if it is not clear which is the direct type of brain tissue damage.

Morphological features indicate that SARS-CoV-2 related coagulation disorders are the main cause of the brain tissue damage. The immediate use of anticoagulant therapy in critical patients reduced thromboembolic complications.

Bacterial superinfection can be the cause of acute leptomeningitis during SARS-CoV-2 infection.

Brain damage can be present even in absence of specific neurological symptoms. Therefore, it is possible that brain involvement could be an underestimated feature in SARS-CoV-2 infected patients [48].

Astrogliosis, microglial activation and chronic inflammation could play a role for the development of chronic neurological disorders.

## Figures and Tables

**Table 1:** Summary of findings in the three pandemic waves.

	First Pandemic Wave (February 2020–May 2020)	Second Pandemic Waves (September 2020–December 2020)	Third Pandemic Waves (Jenuary 2021–April 2021)
**Patients Cases**	11	9	13
**Clinical data**			
Mean age (years)	60.6	65.6	68.1
Range (years)	44–74	47–79	58–90
*Gender*			
*M*	8 (72%)	6 (54%)	11 (84%)
*F*	3 (28%)	3 (46)	2 (16%)
Syntomps duration before	22	25	20
death (days)			
Range	6–51	6–45	5–43
**Gross features**			
Infarction	3 (27%)	1 (11%)	1 (7%)
*Parenchymal hemorrhage*	0 (0%)	0 (0%)	1 (7%)
Herniation	2 (18%)	1 (11%)	1 (7%)
*Meningeal vessel congestion*	11 (100%)	9 (100%)	9 (69%)
Meningeal hemorrhage	1 (9%)	0 (0%)	1 (7%)
*Meningitis*	3 (27%)	0 (0%)	0 (0%)
**Microscopic features**			
Small vessels ectasia, variable perivascular edema, microhemorrhages	11 (100%)	9 (100%)	13 (100%)
*Global hypoxic-ischemic injury and microglial activation*	11 (100%)	9 (100%)	13 (100%)
Microthrombi	11 (100%)	6 (66%)	3 (23%)
*Recent microinfarcts*	9 (81%)	2 (22%)	2 (15%)
Meningeal chronic lymphocytic inflammation	1 (9%)	5 (55%)	9 (69%)

## Data Availability

All the data that have been cited in this paper are openly available in PubMed^®^ at https://pubmed.ncbi.nlm.nih.gov/ (accessed on 1 April 2022). All the data supporting the findings of this study are available from the corresponding author on request.

## Supplementary Materials

The following supporting information can be downloaded at: https://www.mdpi.com/article/10.3390/biom12050629/s1: Table S1: clinical data, Table S2: Summary of gross neuropathological findings, Table S3: summary of histological findings.
