# Supplementary material for: COVID-19 and the Brain: The Neuropathological Italian Experience on 33 Adult Autopsies
Source: Biomolecules. 2022 Apr 25;12(5):629. doi: 10.3390/biom12050629 (PMC9138268; doi:10.3390/biom12050629)
Supplement: Supplementary file 1 [file biomolecules-12-00629-s001.zip › Table 1 Supp File 15Ap.pdf]

Table S1: clinical data (M: male; F: female; ECMO: Extra-Corporeal Membrane Oxygenation; BAL: Broncho-Alveolar Lavage; PNX: pneumothorax; OB: obstructive bronchitis; MRSA: Methicillin-resistant Staphylococcus aureus; UIP: usual interstitial pneumonia; DVP: deep vein thrombosis; DAOD: distal arterial occlusive disease, RBBB: Right bundle branch block).

		Age	*Gender*	*Symptoms duration before death (days)*	*PM interval (hours)*	*Associated pathologies*	*First symptoms*	*Other*
First pandemic wave <br>***(02/2020-04/2020)***	Case 1	51	M	6	31	- Ictus cerebri (2006)<br>- Hypertension<br>- Glaucoma<br>- Kidney failure<br>- Drug abuser	- Dyspnea<br>- Fever	- Dialysis<br>- *C. glabrata*
	Case 2	64	M	16	72	- Obesity<br>- Hypertension	- Dyspnea<br>- Fever	- Dialysis
	Case 3	70	F	13	38	- Ictus cerebri (2015)<br>- Obesity<br>- Smoker	- Dyspnea<br>- Fever	
	Case 4	62	M	14	36	- Obesity<br>- Hypertension	- Dyspnea<br>- Fever	
	Case 5	44	M	26	29	- Diabetes type I<br>- Obesity<br>- Hypertension	- Dyspnea	- ECMO<br>- *E. coli*
	Case 6	64	F	25	50	- Obesity<br>- Crohndisease	- Dyspnea	- *C. glabrata*
	Case 7	52	M	16	55	- Obesity<br>- Hypertension	- Dyspnea	
	Case 8	66	M	35	25	- Hypertension<br>- Dyslipidemia	- Fever	- *P. aeruginosa*<br>- *S. aureus*<br>- *C. albicans*<br>- *S. capitis*
	Case 9	74	M	26	24	- Ischemic cardiomyopathy (previous acute myocardial infarction)<br>- OB<br>- Atrial fibrillation<br>- Diabetes type II	- Urinary retention (pelvic mass)<br>- Fever<br>- Syncope<br>- Previous pneumonia (February)	- During hospitalization, pneumonia SARS-CoV2 related<br>- *MRSA*<br>- PNX
	Case 10	62	F	17	37	- Hypothyroidism	- Fever<br>- Muscular weakness	- *S. aureus*

	Case 11	58	M	51	24	- Hypertension	- Fever<br>- Dyspnea	*- E- faecium*<br>*- S. capitis*<br>*- P. aeruginosa*
Second pandemic wave<br><br>***(09/2020-12/2020)***	Case 12	63	F	30	24	- Hypertension<br>- Dyslipidemia		
	Case 13	79	F	9	24	- Obesity<br>- Hypertension	. Sepsis	*-DVP*
	Case 14	66	M	38	24	- Obesity<br>- OB<br>- Hypertension	- Fever<br>- Dyspnea	*- ECMO*<br>*- multiple bleedings*
	Case 15	52	F	6	24	- Kidney failure<br>- Multiple Myeloma<br>- Rheumatoid arthritis	- Dyspnea<br>- low back pain	
	Case 16	74	M	31	24	- Hypertension<br>- Dyslipidemia<br>- UIP	- Fever<br>- Dyspnea	*- Aspergillus*
	Case 17	75	M	36	24	- Hypertension	- Fever<br>- Dyspnea	*- S. epidermidis*<br>*- S. pneumoniae*
	Case 18	58	M	10	24	- Hypertension<br>- Dyslipidemia<br>- Obesity<br>- Diabetes type II	- Fever<br>- Dyspnea	
	Case 19	77	M	45	24	- Colonic adenocarcinoma<br>- Dyslipidemia<br>- Obesity<br>- Diabetes type II	- Urinary Sepsis<br>- Dyspnea	*- Acute cholecystitis*<br>*- E- faecium*
	Case 20	47	M	25	24	- Obesity	- Fever<br>- Syncope	*- K.pneumoniae*<br>*- Aspergillus*
Third pandemic wave<br><br>***(01/2021-04/2021)***	Case 21	86	M	11	24	- DAOD<br>- Kidney failure<br>- Coronaropathy<br>- Dyslipidemia<br>- DM	- Fever and SARS-CoV-2 positivity after surgery (DAOD)	*- S. lugdunensis*
	Case 22	55	F	12	72		- Syncope	*- DVP and pulmonary embolism*
	Case 23	60	M	43	72	- Hypetension	- Fever and SARS-CoV-2 positivity after surgery (gallbladder)	*- Acinetobacter*<br>*- K. pneumonia*<br>*- Cerebral bleeding*
	Case 24	59	M	10	24	- Obesity<br>- Hypertension	- Dyspnea	
	Case 25	72	M	14	72	- Renal Cell Carcinoma<br>- Colon Cancer	- Fever<br>- Dyspnea	*- Retroperitoneal haematoma*
	Case 26	75	F	24	48	- Obesity<br>- Hypertension	- Fever<br>- Weakness	*- E. faecalis*

					- Dyslipidemia		
Case 27	59	M	28	24	- Coronaropathy<br>- Hypetension<br>- RBBB	- Dyspnea<br>- Weakness	*- P. aeruginosa*
Case 28	78	M	27	48	- OB	- Fever<br>- Dyspnea	*- S.epidermidis*
Case 29	58	M	27	24	- Smoker	- Dyspnea<br>- Headache	*- DVP*
Case 30	66	M	18	24	- Obesity<br>- Hypertension<br>- DM	- Fever<br>- Dyspnea	
Case 31	63	M	10	24	- Obesity<br>- Hypertension<br>- Ischemic cardiomyopathy (previous acute myocardial infarction)	- Fever<br>- Dyspnea	*- Kidney failure*
Case 32	65	M	19	24	- DM<br>- Dyslipidemia<br>- Kidney failure	- Fever<br>- Dyspnea	
Case 33	90	M	5	24	- DM<br>- Kidney failure	- Fever<br>- Dyspnea	
