# Supplementary material for: COVID-19 and the Brain: The Neuropathological Italian Experience on 33 Adult Autopsies
Source: Biomolecules. 2022 Apr 25;12(5):629. doi: 10.3390/biom12050629 (PMC9138268; doi:10.3390/biom12050629)
Supplement: Supplementary file 1 [file biomolecules-12-00629-s001.zip › Table 2 Supp File 15 Apr.pdf]

Table S2: Summary of gross neuropathological findings (*IC: internal carotid*).

		**Brain features**				**Meningeal features**			**Large vessels**
		Weight (gr.)	*Oedema*	*Infarction/Haemorrhage*	*Herniation*	*Vessels congestion*	*Blood extravasation*	*Purulent exudate*	
First pandemic wave (02/2020-04/2020)	***Case 1***	1480	Yes	Left frontal infarction		Yes			*Atherosclerosis*
	Case 2	1670	Yes	Right parietal infarction		Yes			
	Case 3	1320	Yes	Right frontal infarction		Yes			
	Case 4	1870	Yes			Yes			
	Case 5	1870	Yes		Uncal	Yes			
	Case 6	1350	Yes		Uncal	Yes			
	Case 7	1650	Yes			Yes			
	Case 8	1300	Yes			Yes		Yes	
	Case 9	1490	Yes			Yes	Yes (supratentorial)	Yes	*Atherosclerosis*
	Case 10	1350	Yes			Yes			
	Case 11	1380	Yes			Yes		Yes	
Second pandemic waves (11/2020-12/2020)	***Case 12***	1360	Yes			Yes			
	Case 13	1390	Yes		Uncal	Yes			
	Case 14	1380	Yes			Yes			
	Case 15	1350	Yes			Yes			
	Case 16	1400	Yes			Yes			
	Case 17	1360	Yes			Yes			

	Case 18	1360	Yes			Yes		*Right IC aneurysm*
	Case 19	1360	Yes	Right pre-rolandic infarction		Yes		*Atherosclerosis*
	Case 20	1390	Yes			Yes		
Third pandemic waves (01/2021-04/2021)	***Case 21***	1680	Yes	Right frontal, pre-rolandic infarction		Yes		*Atherosclerosis*
	Case 22	1290	Yes			Yes		
	Case 23	1580	Yes	Multiple acute haemorrhages (brain, brain stem, cerebellum)		Yes	Yes (tentorium)	*Atherosclerosis*
	Case 24	1420	Yes			Yes		
	Case 25	1230	Yes			Yes		
	Case 26	1150	Yes			Yes		
	Case 27	1450	Yes			Yes		
	Case 28	1360	No			No		
	Case 29	1600	Yes		Uncal	Yes		*Atherosclerosis*
	Case 30	1400	Yes			Yes		*Atherosclerosis*
	Case 31	1290	Yes					
	Case 32	1400	Yes					
	Case 33	1410	Yes					
