# Supplementary material for: COVID-19 and the Brain: The Neuropathological Italian Experience on 33 Adult Autopsies
Source: Biomolecules. 2022 Apr 25;12(5):629. doi: 10.3390/biom12050629 (PMC9138268; doi:10.3390/biom12050629)
Supplement: Supplementary file 1 [file biomolecules-12-00629-s001.zip › Table 3 Supp File 15 apr.pdf]

Table S3: summary of histological findings.

		SARS CoV-2 RNA SNC	Brain features					Leptomeningeal features		
			Global hypoxic-ischemic injury and microglial activation	***Small vessels ectasia, variable perivascular edema, microhaemorrhages***	***Microthrombi***	***Recent microinfarcts***	***Ancient infarct***	***Chronic lymphocitic inflammetion***	***Blood extravasation***	***Purulent exudate***
First pandemic wave (02/2020-04/2020)	***Case 1***	X olfactory tract, medulla oblongata)	X	X	X	Cortical	Frontal lobe			
	Case 2		X	X	X	Cortical	Parietal lobe			
	Case 3		X	X	X	Cortical and basal ganglia	Frontal lobe			
	Case 4		X	X	X	Cortical		X		
	Case 5		X	X	X	Basal ganglia, brain stem				
	Case 6		X	X	X	Cortical				
	Case 7		X	X	X	Cortical				
	Case 8		X	X	X	Cortical, brain stem				X
	Case 9		X	X	X				X	X
	Case 10		X	X	X	Para-hippocampus				
	Case 11		X	X	X					X
Second pandemic	***Case 12***		X	X	X Rare			X		
	Case 13		X	X						
	Case 14		X	X						

wave ***(11/2020-12/2020)***	***Case 15***		X	X			Skull and dural plasmocytoma	
	Case 16		X	X	X Rare			
	Case 17		X	X	X Rare		X	
	Case 18		X	X	X	Parietal, brain stem, pituitary gland	X	
	Case 19		X	X	X Rare	Rolandic	X	
	Case 20	X (olfacrtory tract)	X	X	X Rare		X	
Third pandemic waves ***(01/2021-04/2021)***	***Case 21***		X	X	X	Frontal, rolandic	X	
	Case 22		X	X				
	Case 23		X	X acute small and large haemorrhages	X	Frontal	X	X (infratentorial)
	Case 24		X	X			X	
	Case 25		X	X			X	
	Case 26		X	X			X	
	Case 27		X	X			X	
	Case 28		X	X	X		X	
	Case 29		X	X			X	
	Case 30		X	X			X	
	Case 31		X	X				
	Case 32		X	X				
	Case 33		X	X				
